# A Bradycardia-Based Stress Calculator for the Neonatal Intensive Care Unit: A Multisystem Approach

**DOI:** 10.3389/fphys.2020.00741

**Published:** 2020-06-26

**Authors:** Mario Lavanga, Bieke Bollen, Katrien Jansen, Els Ortibus, Gunnar Naulaers, Sabine Van Huffel, Alexander Caicedo

**Affiliations:** ^1^Division STADIUS, Department of Electrical Engineering (ESAT), KU Leuven, Leuven, Belgium; ^2^Department of Development and Regeneration, Faculty of Medicine, KU Leuven, Belgium; ^3^Applied Mathematics and Computer Science, School of Engineering, Science and Technology, Universidad del Rosario, Bogotá, Colombia

**Keywords:** preterm infants, perinatal stress, pain, bradycardia, desaturation, network physiology, EEG, HRV

## Abstract

Early life stress in the neonatal intensive care unit (NICU) can predispose premature infants to adverse health outcomes and neurodevelopment delays. Hands-on-care and procedural pain might induce apneas, hypoxic events, and sleep-wake disturbances, which can ultimately impact maturation, but a data-driven method based on physiological fingerprints to quantify early-life stress does not exist. This study aims to provide an automatic stress detector by investigating the relationship between bradycardias, hypoxic events and perinatal stress in NICU patients. EEG, ECG, and *SpO*_2_ were recorded from 136 patients for at least 3 h in three different monitoring groups. In these subjects, the stress burden was assessed using the Leuven Pain Scale. Different subspace linear discriminant analysis models were designed to detect the presence or the absence of stress based on information in each bradycardic spell. The classification shows an area under the curve in the range [0.80–0.96] and a kappa score in the range [0.41–0.80]. The results suggest that stress seems to increase *SpO*_2_ desaturations and EEG regularity as well as the interaction between the cardiovascular and neurological system. It might be possible that stress load enhances the reaction to respiratory abnormalities, which could ultimately impact the neurological and behavioral development.

## 1. Introduction

Premature infants are at risk of maladaptive outcomes and neurodevelopment delays. Patients who spend their early life in the neonatal intensive care unit (NICU) can undergo profound alterations of sleep-patterns as well as exposure to painful procedures and noxious stimuli (Grunau, 2013; Barbeau and Weiss, 2017). Grunau (2013) have shown how stress exposure can induce a cascade of physiological consequences, behavioral and hormonal responses. In addition, Brummelte et al. (2012) highlighted how procedural pain can affect structural connectivity of the subcortical areas during neurodevelopment.

In particular, routine day-care has been reported to affect sleep quality inside the NICU (Barbeau and Weiss, 2017). Levy has shown that prolonged contact in NICU can have multiple consequences. 57% of the sleeping infants experience awakening because of hands-on care. Handling is usually followed by respiratory events, such as hypoapneas and apneas, or desaturations. Surprisingly, clinical handling is more likely to initiate oxygen desaturation and bradycardias. Monitoring of respiratory and hypoxic events is pivotal since experience of long bradycardias and apneic spell in very-low weight infants are known to impact the development of the patients (Pichler et al., 2003; Janvier et al., 2004; Horne et al., 2017). In particular, Janvier et al. (2004) have shown that a higher apnea burden (total amount of apnea days in the ward) is associated to a worsening of the cognitive and motor outcome. Prolonged oxygen desaturations associated with bradycardias are known to have greater negative effect on cerebral oxygenation (Pichler et al., 2003) and the persistence of their effect can be even prolonged at 5–6 months corrected age, with worse *SpO*_2_ and heart-rate drops compared to full-term infants at equivalent age (Horne et al., 2017). Furthermore, bradycardias were under scrutiny in different studies as sign of autonomic nervous system development. Gee et al. (2017) has shown how the heart-rate variance and entropy dramatically change before any heart-rate drop. This could be the consequence of a dysfunction of vagal stimulation, which induces the bradycardia, according to the polyvagal theory by Porges. Those events are usually preceded by low-heart rate variability as sign of fetal distress (Porges, 1995).

Although a possible link exists between stress burden and cardiorespiratory events, an automated method to quantify stress exposure in the NICU based on physiological signal activity, especially during oxygen desaturations or bradycardias, has not been described yet. However, the literature provides an overview how physiological signals can be used to investigate pain and apneic spells in adults. Multiple authors described machine-learning models to classify pain-patterns using different modalities, such as EEG or EMG (Gruss et al., 2015; Misra et al., 2017). In parallel, other authors described an algorithm to detect apnea events based on *SpO*_2_ analysis (Deviaene et al., 2018). In addition, some authors have already investigated a possible link between modalities that describe brain activity and modalities that describe cardiovascular activity in the case of apneic spells or desaturation events. Specifically, a recent study proposed a model to explain how pre-frontal cortex dysfunctions in adults and children can be caused by obstructive sleep apneas due to disruption of sleep and chemical homeostasis (Beebe and Gozal, 2002). Pitson et al. showed that *SpO*_2_ dips due to apneas are related to the patients' daily sleepiness, which can affect the emotional and behavorial state. Interestingly, those desaturation events seem to significantly correlate to other physiological events, such as EEG and heart-rate arousals (Pitson and Stradling, 1998).

This inherent coordination of different physiological systems in case of apneas, as highlighted by Pitson and Stradling (1998), or the necessity to rely on different modalities to classify biopotential information, as shown by several authors (Gruss et al., 2015; Misra et al., 2017), strongly suggest a horizontal interaction among organs, which might be altered in case of stress or hypoxia and might require different tools to approach the alteration of the physiological state of the patients (Ivanov et al., 2016). This synchronization among different organs or signal modality is known as Network Physiology and was specifically applied to show the alteration between brain activity and parasympathetic tone of the HRV (Jurysta et al., 2006) and the synchrony between the neonatal EEG bursts and the heart-rate accelerations of the infants (Pfurtscheller et al., 2008). However, one might investigate network physiology in the infants and relate that to a specific physiological condition. As highlighted by Bashan et al. (2012), physiological systems under neural regulation exhibit a high degree of complexity with non-stationary, intermittent, scale-invariant and non-linear behavior and change in time under different physiological states and pathological conditions. One can not only simply derive the integration among the different physiological systems, but might also try to summarize the topological properties of the physiological network and investigate their evolution over time (Bartsch and Ivanov, 2014; Bartsch et al., 2015). The clinical literature also suggested that the overall activity of the individual physiology cannot simply be summarized as the sum of the individual organs' physiology, but it requires an investigation of the interaction among the different sub-systems, especially in the intensive care setting (Moorman et al., 2016).

Since the clinical literature has already shown a unique relationship between handling of infants and apneas or hypoxic events, the aim of this study is the development of a classification model to relate hypoxias to patient's stress exposure. A binary classifier was developed to classify whether a bradycardic event belonged to a patient with stress or without stress burden. Due to the interdisciplinary nature of hypoxic events and stress exposure, the study aimed not only to derive the features from different modalities, but assess the network physiology of the patients and its relationship with stress load and bradycardias. In this article, stress is defined as accumulation of procedural pain, based on a previous study (Grunau, 2013). The paper is organized as follows: in section Material and Methods, the data collection and stress classification models are outlined. In section Results, the results of the study are presented, while the last section focuses on the implication of this research.

## 2. Materials and Methods

### 2.1. Patient Sample

Data from pre-term infants were collected as part of the Resilience Study, which has been carried out in the Neonatal Intensive Care Unit (NICU) of the University Hospitals Leuven, Belgium. Parents of pre-term infants born before 34 weeks gestational age (GA) and/or with a birth weight <1,500 g were approached for informed consent within the first 3 days after birth. A total of 136 patients was included in the cohort from July 2016 to July 2018. Exclusion criteria were as follows: parents' age <18 years, limited knowledge of Dutch or English, medical (somatic or psychiatric) condition in the parent(s) that impeded participation, and the presence of a major congenital malformation or central nervous system pathology (grade 3 or grade 4 intraventricular hemorrhage or periventricular leukomalacia) at the time of consent.

The research protocol has been examined and approved by the Ethical Committee of University Hospitals Leuven, Belgium. The Resilience Study was performed in accordance with the Guidelines for Good Clinical Practice (ICH/GCP) and the latest version of the Declaration of Helsinki. It has been registered at Clinical Trials.gov (NCT02623400).

### 2.2. Data Acquisition

During the NICU stay, pain levels were daily recorded with a multidimensional scale for premature infants known as the Leuven Pain Scale (LPS). This scale varies in the range [0,14] and is obtained as the sum derived by seven categories (such as crying, grimace or heart-rate) (Allegaert et al., 2003, 2013). LPS scores were routinely daily recorded by bed-side nurses, every hour for the intensive care patients and every 3 h for the intermediate care.

Based on the association between stress and pain, perinatal stress has been defined as the presence of non-zero LPS in the patient record the day before the recording, i.e., *LPS* > 0, which means experience of any pain the day before the recording.

According to the clinical protocol, EEG, ECG, and *SpO*_2_ measurements were recorded for at least 3 h in three monitoring groups: the first measurement took place around 5 days after birth (5days), while the second and the third recording were respectively planned around 34 weeks post-menstrual age (PMA) (34w) and in the week before discharge home. The last recording usually consisted of a 24 h polsomnography, therefore the last group was labeled as PSG. Only one of the first two recordings was performed for infants born at 33–34 weeks. In the course of their NICU stay, some infants were transferred to level II units in hospitals closer to home. Therefore, not all infants have multiple recordings and some LPS measures are missing. A total of 245 recordings had corresponding pain scores available and were analyzed. A total of 39 patients had three recordings with associated pain score. A set of 38 patients had two recordings and the remaining 52 had 1 recording (39 * 3 + 38 * 2 + 52 = 245). Table 1 summarizes the clinical characteristics of patients at each measuring point. EEG set-up included nine monopolar electrodes (*F*_*p*_1, *F*_*p*_2, *C*_3_, *C*_4_, *C*_*z*_, *T*_3_, *T*_4_, *O*_1_, *O*_2_) and the EEG signals were referenced to the electrode *C*_*z*_. The sampling frequencies for EEG, ECG and *SpO*_2_ were 256, 500, and 1 Hz, respectively. They were monitored with the OSG system (OSG BVBA, Brussel). The R-peaks of the ECG were detected via the R-DECO toolbox (Moeyersons et al., 2019) and the tachogram or HRV signal was derived as subsequent R-peak to R-peak intervals (*RR*_*i*_).

**Table 1:** Summary of patient data demographics at different time points: GA (gestational age), birth weight (in g), PMA (post-menstrual age) at EEG and ECG recording, LPS (Leuven Pain Score).

	**5 days (*n* = 118)**	**34 weeks (*n* = 67)**	**PSG (*n* = 117)**
GA (weeks)	31.14 [28.86–32.43]	28.86 [26.86–30.71]	30.29 [27.29–31.71]
Birth weight (g)	1475 [1120–1725]	1140 [900–1480]	1225 [950–1540]
PMA (weeks)	32.14 [30–33.43]	34.14 [33.86–34.29]	38.43 [37.29–39.57]
LPS	1 [0–3]	0 [0–2]	0 [0–2]

*Data is reported as q_50_[q_25_–q_75_], where q_50_ is the median and q_25_–q_75_ is the IQR*.

### 2.3. Bradycardia Detection and Data Pre-processing

Multiple studies have shown that hands-on-care and clinical handling can disrupt the sleep cycle and induce oxygen desaturations and apneic spell (Barbeau and Weiss, 2017; Levy et al., 2017). The most threatening desaturations for the brain physiology and the development of the infant are usually events concurrent with bradycardia, i.e., a sudden drop in heart-rate (Pichler et al., 2003; Horne et al., 2017). Since Levy et al. (2017) has shown that bradycardias, apneas, hypoapneas, and hypoxic events are linked to stress exposure and Porges (1995) relates bradycardias to fetal distress, the definition of apnea prematurity was followed to detect cardiorespiratory events or desaturations in the physiological signal (Paolillo and Picone, 2013). Clinically relevant apneas were characterized by RR elongation above $1.5*\bar{RR_{i}}$ for at least 4 s, where $\bar{RR_{i}}$ is the average of the entire tachogram, with a variation of *SpO*_2_ > 10% with respect to the baseline (Janvier et al., 2004). Consequently, hypoxic events were detected as events with concomitant variations of HRV and oxygen saturation, defined by increases above $1.5*\bar{RR_{i}}$ for more than 4 s and *SpO*_2_ desaturations exceeding the following different thresholds: 3%, 5% and 10%. The saturation drops from the baseline were detected according to Deviaene et al. (2018) and the different thresholds were used to test whether stress exposure induces more pronounced hypoxic events. Normally, apneas are defined as breathing cessation for more than 20 s. However, both Barbeau and Weiss (2017) and Levy et al. (2017) have shown that events due to NICU handling are not necessary full apneic spells, but mostly physiological events like hypopneas and desaturations which last shortly and do not reach the level of clinical alarm. Gee et al. (2017) and Porges (1995) outlined the solely and specific importance of bradycardias as sign of dismaturity and distress of the premature infant. In addition, the respiration signal in our study was frequently distorted by artifacts and usually derived from the ECG for the younger patients. Therefore, the event detection specifically targeted bradycardias, instead of looking at the general breathing cessations. For each of those events, a window of 3 min before and after each *bradycardia peak* was the starting interval to develop a stress classifier. Specifically, a bradycardia peak is the moment of maximal heart-rate drop or RR intervals elongation. For each epoch, the EEG signal was filtered between [1–20] Hz and possible EOG artifacts were filtered using independent component analysis.

### 2.4. Features Extraction

Multiple features were extracted from the EEG, HRV, *SpO*_2_ from each bradycardic spell to assess its relationship with stress. They were computed at least in two moments: the period before the bradycardic event, i.e., from the start of the window until the *RR*_*i*_ exceeds $1.5*\bar{RR_{i}}$ threshold, and the period after the bradycardic event, which goes from the moment *RR*_*i*_ comes back to stationarity until the end of the window. According to the different methodologies, features were also computed during the bradycardia or during the entire hypoxic spell. The computation within the bradycardia was not always possible since indices like fractality require higher number of samples that were not available. Furthermore, the epoch durations were variable depending on the length and the intensity of the bradycardic event. An overview of the different features are reported in Tables 2, 3.

**Table 2:** Overview of the univariate features derived from the physiological signal in the study.

	**Temporal**	**Spectral**	**Non-linear**
*HRV*	μ_*RR*_, σ_*RR*_,	μ_*HF*_, σ_*HF*_, μ_*LF*_, σ_*LF*_	*C*_*x*_, *C*_*y*_
	*Slope*_*OV*_(*T*), *Slope*_*AP*_(*T*)	μ_*HF*_*nu*__, σ_*HF*_*nu*__, $\mu_{\frac{LF}{HF}}$, $\sigma_{\frac{LF}{HF}}$	*SD*_1_, *SD*_2_
*SpO*_2_	μ_*SpO*_2__, σ_*SpO*_2__,		*C*_*x*_, *C*_*y*_
	*Slope*_*OV*_(*T*), *Slope*_*AP*_(*T*)		*SD*_1_, *SD*_2_
*EEG*	μ_*EEG*_, σ_*EEG*_,	μ_*P*(δ)_, σ_*P*(δ)_	*H*_*exp,EEG*_, *H*_*exp,P*(δ)_
			*c*_2,*EEG*_, *c*_2,*P*(δ)_

*For each signal (HRV, SpO_2_, EEG), the temporal, spectral and non-linear attributes are reported. The total count for HRV is 26: 2 temporal features, 8 spectral features, 4 non-linear features from the Poincaré plot (PP) and 12 PRSA features. The total count for SpO_2_ is 18: 2 temporal features, 4 non-linear features and 12 PRSA features. The total count for EEG is 64: 2 temporal features, 2 spectral features and 4 fractal features repeated for each channel. RR and P(δ), respectively represent the tachogram or HRV and the EEG power in the δ band. μ and σ stand for mean and standard deviation. LF and HF represent the high and low-frequency bands of HRV. H_exp_ and c_2_ are the main Hurst exponent and the width of the singularity spectrum derived with the multifractality framework. C_xy_ and SD_12_ are the PP features. Slope_OV_(T) represents the overall PRSA slope from the start of its window, while slope_AP_(T) is the slope around each anchor point. Six different window lengths T were selected to define each anchor point: [1,5,10,20,50,100] s*.

**Table 3:** Overview of the multivariate features derived from the different monitoring groups and the possible interaction combinations among the different modalities (EEG-*SpO*_2_, EEG-*RR*_*i*_, EEG-EEG, EEG-*SpO*_2_-*RR*_*i*_).

	***EEG-SpO*_2_**	***EEG-RR*_*i*_**	***EEG-EEG***	***EEG-SpO*_2_ − *RR*_*i*_**
5_*days*_	*Path*_*length*_(*VLF*),	*Path*_*length*_(*VLF*),	*Path*_*length*_(*VLF*),	*Path*_*length*_(*VLF*),
	*Efficiency*(*VLF*),	*Efficiency*(*VLF*),	*Efficiency*(*VLF*),	*Efficiency*(*VLF*),
	*Clust*_*c,node*_(*VLF*),	*Clust*_*c,node*_(*VLF*),	*Clust*_*c,node*_(*VLF*),	*Clust*_*c,node*_(*VLF*),
	*Ecc*_*node*_(*VLF*),	*Ecc*_*node*_(*VLF*),	*Ecc*_*node*_(*VLF*),	*Ecc*_*node*_(*VLF*),
	*n*_*sup*_(*VLF*)	*n*_*sup*_(*VLF*)	*n*_*sup*_(*VLF*)	*n*_*sup*_(*VLF*)
34_*weeks*_,	*Path*_*length*_(*LF*),	*Path*_*length*_(*LF*),	*Path*_*length*_(*LF*),	*Path*_*length*_(*LF*),
*PSG*	*Efficiency*(*LF*),	*Efficiency*(*LF*),	*Efficiency*(*LF*),	*Efficiency*(*LF*),
	*Clust*_*c,node*_(*LF*),	*Clust*_*c,node*_(*LF*),	*Clust*_*c,node*_(*LF*),	*Clust*_*c,node*_(*LF*),
	*Ecc*_*node*_(*LF*),	*Ecc*_*node*_(*LF*),	*Ecc*_*node*_(*VLF*),	*Ecc*_*node*_(*LF*),
	*n*_*sup*_(*LF*)	*n*_*sup*_(*LF*)	*n*_*sup*_(*LF*)	*n*_*sup*_(*LF*)

*For 5_days_, the interaction was derived specifically for the VLF band, while the interaction for other two groups was assessed in LF band. The set of attributes for each monitoring group is 84: 21 features for EEG-SpO_2_, 21 features for EEG-RR_i_, 19 for EEG-EEG, 23 features for EEG-SpO_2_-RR_i_. The clustering coefficient (Clust_c_) and the eccentricity (Ecc) are node-dependent features, which explain the variation in numbers for each interaction group. The actual count rises to 168 since both mean and standard deviation are considered. n_sup_ is the number of superfluous connections. The label Efficiency represents the global efficiency of the network. VLF and LF represent the very-low and low-frequency bands of HRV*.

#### 2.4.1. Cardiovascular Analysis: HRV and *SpO*_2_ Features

The tachogram's reactivity was investigated with classical temporal and spectral indices. Specifically, the power spectral density (PSD) of the tachogram was computed with the continuous wavelet transform using analytical Morlet as mother wavelet. The absolute powers in the high-frequency (HF) and low-frequency (LF) range were derived as sum of the PSD bins in the following frequency bands: *HF* = (0.2 − 4] *Hz* and *LF* = (0.08 − 0.2] *Hz* (David et al., 2007). The indices $\frac{LF}{HF}$ and $\frac{HF}{LF+HF}$ were used to assess the contribution of the multiple autonomic branches (Hoyer et al., 2013). Since the wavelet-approach derives the time-frequency distribution of a signal, both the mean and the standard deviation of those indices, together with the temporal mean and standard deviation of the HRV, were derived as features in the epochs before, during and after each bradycardic spell. Additionally, the heart-beat dynamics were assessed via the Poincaré Plot (PP) analysis. The PP are two-dimensional scatter-plots where *RR*(*t*) is plotted vs. the lagged sample *RR*(*t* + τ). This graphical representation is a simplification of Taken's theorem to represent the phase space in order to assess the non-linear behavior of the signal. The lag τ was estimated as the first zero of the autocorrelation function of the signal and the PP can then be described by the matrix **X** = [*RR*(*t*), *RR*(*t* + τ)], where *RR*(*t*) is a vectorial representation of the HRV time series of dimension ℝ^(*N*−τ)×1^, where N represents the length of the signal. Most commonly, the standard deviations *SD*_1_ and *SD*_2_ of the minor and major axis of the cloud defined by X are computed to represent the short and long-term RR variability. In this study, the information in the PP was quantified via *SD*_2_ and *SD*_1_ as the first two singular values of X and via the centroids *C*_*x*_ and *C*_*y*_ of the same matrix as the column-wise mean of matrix X. The PP was represented and investigated using the entire bradycardic window.

Similarly to HRV, temporal features, such as mean and standard deviation, as well as the PP features were derived from *SpO*_2_. Concerning the epochs for *SpO*_2_ features computation, the epoch before and after *SpO*_2_ dips were considered, i.e., the epoch that starts from the beginning of the window until *SpO*_2_ exceeds the considered threshold and the epoch that starts from the moment that *SpO*_2_ goes back to stationarity until the end of the window.

Desaturation events and bradycardic spells never occur alone, especially when driven by hands-on-care. The periodicity of both *SpO*_2_ dips and heart-rate can be characterized by Phase Rectified Signal Averaging (PRSA), which searches for all time points where the signal goes downward (or upward) in the 6 min segments (Bauer et al., 2006). Fragments of 120 s duration were extracted around each time point, known as anchor point, and they were subsequently aligned and averaged. From this average curve, the overall slope and the slope before and after each anchor point were derived to describe the rate of increase or decrease, such as a desaturation trend or bradycardia increase (Bauer et al., 2006). However, the computed average rate is sensitive to the definition of the anchor points, which ultimately represent an increase or decrease for a specific time window of length T according to the properties of the signal. Therefore, multiple parameters T were investigated in the range [1, 5, 10, 20, 50, 100] s to define the best set of PRSA features.

#### 2.4.2. Neurological Analysis: EEG Features and Multivariate Attributes

Pitson and Stradling (1998) have shown how EEG arousals are related to *SpO*_2_ dips in respiratory events due to obstructive sleep apneas. Those arousals have been defined as an increase in the main carrier frequency of EEG in windows of 10s or more. Furthermore, different authors have shown swings in burst activity as a consequence of HR variations in premature infants (Pfurtscheller et al., 2008; Schwab et al., 2009). The increase in discontinuity and burst-like type of activity are known biomarkers for brain dismaturity or pain elicitation (Fabrizi et al., 2011; Pavlidis et al., 2017). Therefore, multiple features have been computed from the EEG to describe the level of discontinuity in terms of slow-wave persistence, regularity and lack of smoothness (Pavlidis et al., 2017). In addition, the concurrent variations of heart-rate, *SpO*_2_ and EEG were investigated to assess whether they are related to the stress load or not.

#### 2.4.3. EEG Time-Frequency Analysis

The cortical activity was analyzed both in the time and frequency domain. The EEG power in the band δ = (0.5 − 4] *Hz*. was computed via the continuous wavelet transform, using the analytical Morlet as mother wavelet. The reason to focus on the delta band is 2-fold. On the one hand, the δ band represents the sensitive band to pain stimuli and contains the dominant frequency of the neonatal EEG, which is the frequency with the highest power (Wallois, 2010; Abdulla and Wong, 2011; Fabrizi et al., 2011). On the other hand, this frequency band represents subcortical areas, such as the thalamus, which are involved in stress management and autonomic control of the nervous system (Pfurtscheller et al., 2017). Similarly to the cardiovascular variables, the mean and the standard deviation for the EEG and the power in the δ band in each channel was derived for the three epochs around the bradycardic peak.

#### 2.4.4. Multifractality

A more discontinuous EEG signal is characterized by higher regularity or self-similarity. Signals with such property are defined as fractals or scale-free signals. These time series have long-exponentially decaying autocorrelation functions (ACF) or a power-law spectrum, whose rates of decay can be defined by the Hurst exponent (H), which assess the level of similarity (Doret et al., 2015). However, complex and discontinuous signals can vary in fractal properties over time, i.e., the Hurst exponent and therefore the rate of ACF decay can differ (Jaffard et al., 2007). Wendt proposed an efficient way to estimate the different fractal properties based on wavelet leaders (Doret et al., 2015). His method estimates the spectrum of singularities *D*(*h*) (SS), which measures the different Hurst Exponents in the signal and the associated fractal dimension (Wendt et al., 2007).

The most interesting attributes of the singularity spectrum are the location of the maximum and its width which are usually defined as *c*_1_, *c*_2_ (Jaffard et al., 2007). According to Jaffard et al. (2007), *c*_1_ is usually considered the main Hurst exponent (*H*_*exp*_) of the multifractal signal, while *c*_2_ is a variational index to represent the amount of fractals inside the signal. Wendt et al. (2007) reported further details of the methodology and of the WLBFM toolbox implemented in MATLAB to estimate the fractal parameters. The parameters *c*_1_, *c*_2_ were estimated for each EEG channel and the associated δ oscillations.

#### 2.4.5. Multivariate Analysis: Brain-Heart Interactions

The interaction among the cortical activity and the cardiovascular variables can be estimated with the time-frequency coherence between the δ oscillations derived with CWT, the HRV and the *SpO*_2_ (Piper et al., 2014). In order to match the temporal scale, all signals were resampled at 8 Hz. The continuous wavelet coherence is then computed as the following ratio:

(1) $$\begin{array}{l} C_{x_{i}↔x_{j}}\left(t,f\right)=\frac{s_{x_{i}↔x_{j}}\left(t,f\right)}{s_{x_{i}}\left(t,f\right)s_{x_{j}}\left(t,f\right)}, \end{array}$$

where *s*_*x*_*i*_↔*x*_*j*__(*t, f*) is the cross-scalogram between the signal *x*_*i*_ and *x*_*j*_, *s*_*x*_*i*__(*t, f*) and *s*_*x*_*j*__(*t, f*) are the autoscalogram of the signals. The signal *x*_*i*_ can be the delta oscillation of an EEG channel, HRV or the *SpO*_2_. The wavelet transform was computed with analytic Morlet as mother wavelet and the coherence was investigated in the very-low-frequency band *VLF* = (0.033 − 0.08] *Hz* in the 5 days group and the low-frequency band LF in the 34 weeks group and the PSG group. As discussed in previous studies (Hoyer et al., 2013; Lavanga et al., 2019), this shift in frequency band is due to undergoing maturation of the autonomic nervous system. The coupling was derived as the maximum absolute imaginary part of *C*_*x*_*i*_↔*x*_*j*__ in the band of interest Lavanga et al. (2018). The statistical validity of each coupling was then tested with amplitude adjusted Fourier transform (AAFT) surrogates. Specifically, each coupling must be greater in value than the coupling estimated for 19 surrogates, in order to guarantee a level of statistical significance α = 0.05. However, due to the large number of channels and exponential number of associations, the pairwise coupling risks to produce collinear features for stress discrimination. Therefore, topological indices were derived via graph theory. The structure of a graph is defined by a set of nodes, that corresponds to one particular signal or specific information derived from a signal (like the power in a specific band). A link is then defined among nodes in case of a significant interaction and a weight value is associated to indicate the strength of the coupling. A weighted graph is then represented by an adjacency matrix A, whose entries *A* = *A*_*ij*_ represent the coupling between nodes i and j (Bullmore and Sporns, 2009). More precisely, *A*_*ij*_ = *C*_*x*_*i*_↔*x*_*j*__, where *C*_*x*_*i*_↔*x*_*j*__ is the general coupling intensity and *i, j* = 1, .., *M*, with M as the total number of signals. Since the direction of interaction is not specified (as underlined by *x*_*i*_ ↔ *x*_*j*_), *A*_*ij*_ is symmetric and its entries represent statistical correlations without any specific direction. In order to describe the graph topology, a list of topological indices have been introduced, such as the path length, the clustering coefficient and the eccentricity (Bondy and Murty, 1976; Bullmore and Sporns, 2009). The path length is the average shortest path to reach a graph node from any other one. The eccentricity of a node represents the maximum distance from one network node to any other, while the clustering coefficient is defined as the average of all weighted triangles around a node. In addition, a graph can risk to be redundant and superfluous connections can emerge as significant, even after surrogate testing (Peters et al., 2013). The capacity of the network to keep the global connectivity in case of connections removal is known as resilience, which can be computed as the number of superfluous connections. Suppose that all couplings of *A* = *A*_*ij*_ are ordered in descending order and the set of original weights of A is defined as $w_{ij}^{0}$. The number *n*_*s*_*up* of superfluous connections is derived as the number that maximizes the following quantity

(2) $$\begin{array}{l} \underset{n}{\max}H\left(w_{ij}\left(n\right)\right)+E\left(w_{ij}\left(n\right)\right)=-\underset{ij}{\sum }w_{ij}\left(n\right)\log\left(w_{ij}\left(n\right)\right)\\ +\underset{ij}{\sum }{\left(w_{ij}\left(n\right)-w_{ij}^{0}\right)}^{2} \end{array}$$

where *H*(*w*_*ij*_(*n*)) is the entropy of the matrix A where n weights were removed. The value *w*_*ij*_(*n*) represents the remaining non-zero weights, while *E*(*w*_*ij*_(*n*)) is the squared error between the new matrix A and the original matrix. In general, a higher redundant network will have a higher *n*_*s*_*up*, since the superfluous connections represent the removed connections to maintain the global connectivity high without deviation from the original matrix. In this study, graph theory was applied as follows: EEG delta oscillations (8 channels), HRV and *SpO*_2_ were involved in the analysis as nodes setting the number M of processes to 10. Since the interaction estimation is based on wavelet coherence, the adjacency matrix was computed for each time sample and therefore it was possible to derive the charts of the different topological indices. The average and the standard deviation of each topological feature was computed before, during and after each bradycardic spell. In order to test the contribution of a specific modality or signal to the stress classification, graph theory indices were not only computed for the entire set of processes, but we used also partitions of the adjacency matrix *A*_*ij*_. Specifically, we considered connections only related to EEG channels (EEG-EEG), the connections between EEG channels and *SpO*_2_ (EEG-*SpO*_2_), the connections between EEG channels and *RR*_*i*_ (EEG-*RR*_*i*_) and the entire set of connections (EEG-*SpO*_2_-*RR*_*i*_), as reported in Table 3. For each of those partitions, the described list of topological indices was computed.

### 2.5. Bradycardia-Based Classification

A customized software tool was developed with MATLAB libraries to detect whether each bradycardic event belonged to a patient with or without stress burden. In summary, the following groups of features were derived for each hypoxic event:

- Temporal and periodicity features: 14 features in total for HRV, 14 features for *SpO*_2_ and 16 features for the EEG
- Spectral features for both HRV and EEG: 8 features for HRV and 16 features for EEG
- Non-linear features: 4 features for HRV, 4 features for *SpO*_2_ and 32 features for EEG
- Brain-heart connectivity topological indices: 168 features in total for HRV, EEG and *SpO*_2_.

A complete overview is reported in Tables 2, 3. Given the fact that features were derived for three epochs (before, during and after each bradycardia), the total number of extracted features was 748.

The power-features were log-transformed. The study investigated whether there was any association between those features and the bradycardic spell in a patient with a stress exposure in the NICU. As mentioned earlier, the presence of stress was defined as experience of pain the day before the recording (*LPS* > 0). However, Gruss et al. have shown that more intense pain can be discriminated in an easier way (Gruss et al., 2015). On top of that, there is no clear consensus on the level of desaturation that can be considered threatening for premature infants (Janvier et al., 2004; Poets, 2010; Levy et al., 2017). Therefore, different levels of hypoxia were tested in the classification, i.e., *SpO*_2_ > 3%, *SpO*_2_ > 5%, *SpO*_2_ > 10%.

The objective of the classification was to discriminate whether a bradycardic event belonged to a patient with or without stress. After testing different classification algorithms, such as support vector machines (SVMs) and linear discriminant analysis (LDA), a classifier based on subspace ensemble with LDA has been designed to separate bradycardic spell in two groups (Ho, 1998). Subspace LDA is an ensemble method like random forest, where the bagging process (random subsampling of the training set) is performed together with a random subsampling of the features to find the best feature subsets to separate the data (Ho, 1998). The clear advantage is to span a greater number of features and allow the model to tune for the best subset. The model was tested according to a leave-one-patient-out (LOPO) scheme for each monitoring group (5 days, 34 weeks, PSG), which meant that all bradycardic event belonging to one patient were put in the test set. The tuning in training-set followed a 10-fold cross-validation and the following set of performance indices were derived each monitoring group: the area under the curve (AUC) and Cohen's kappa between machine learning labels and the clinical labels. It is important to remind the only one set of indices was obtained for each classifier since they were obtained by pooling all test sets of the different patients together.

Given that the number of features should be below one tenth of the training dataset, the subspace of features has been restricted to 1/10 (one-tenth) of the data (Misra et al., 2017). However, before tuning of the model, features were further reduced before the subspace ensemble algorithm was applied. The considered attributes had intra-feature correlation below 90% and the highest F-scores. The F-score is a simple measure to assess the discrimination between the positive and the negative class. It is computed as the ratio between the separation between positive and negative class (intra-class variability) and the separation within each class (inter-class variability). The details of the procedure are reported here (Chen and Lin, 2006). In addition, the features were corrected by the baseline effect of age in case subject's PMA was a covariate of the feature of interest (i.e., significant Pearson correlation or *p* < 0.05).

## 3. Results

**Figure 1:**
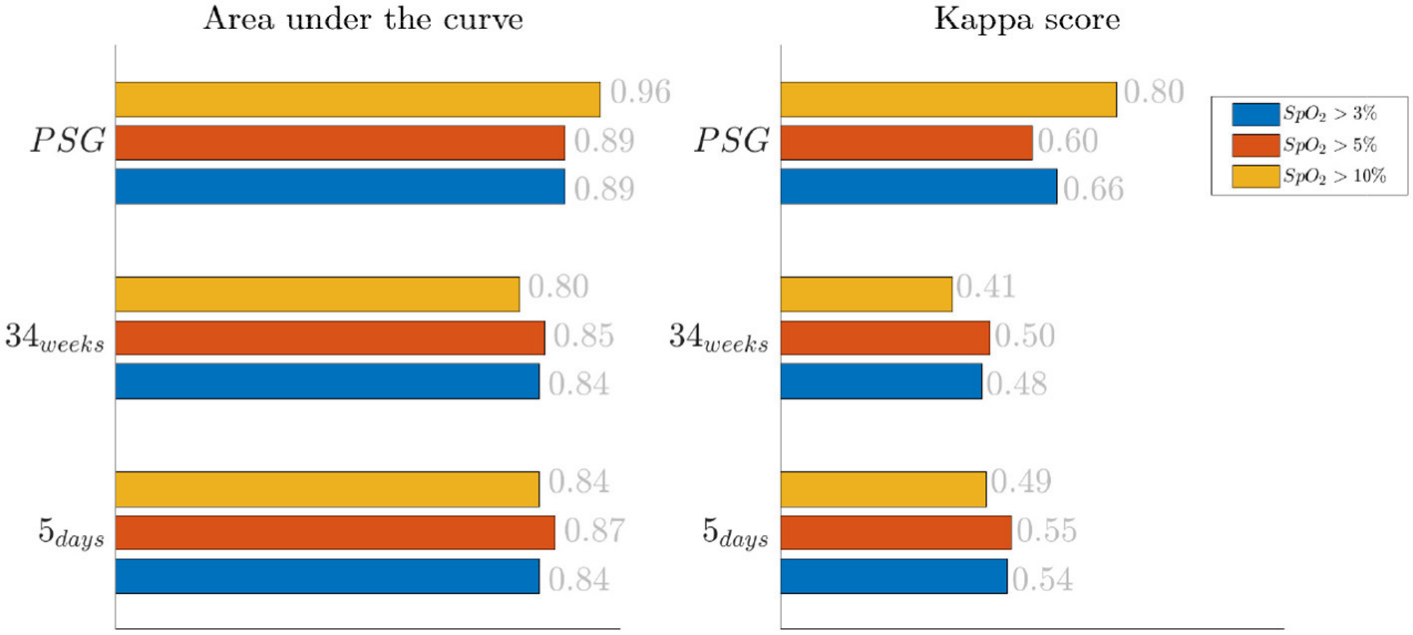
Results of the bradycardia-based classification in three main datasets. The three colors represent different levels of desaturation to consider the bradycardic event in the stress classification. The left panel displays the *area under the curve* in the three monitoring groups, while the right reports Cohen's kappa.

The results for the bradycardia-based stress classification are reported for the three monitoring groups in Figure 1. The AUC and kappa scores are reported in function of the desaturation threshold used to define which events should have been included in the classifier. Each color represent a threshold: blue for desaturations higher than 3%, yellow for desaturations higher than 5% and red for desaturations higher than 10%. The results suggest a moderate association between the bradycardia features and the clinical labels: the AUC lies in the range [0.80–0.96] and the kappa score lies in the range [0.41–0.80]. The *SpO*_2_ threshold for the desaturation seems to have a mild effect on classification: only the PSG group reports an increasing Kappa score for higher threshold.

**Figure 2:**
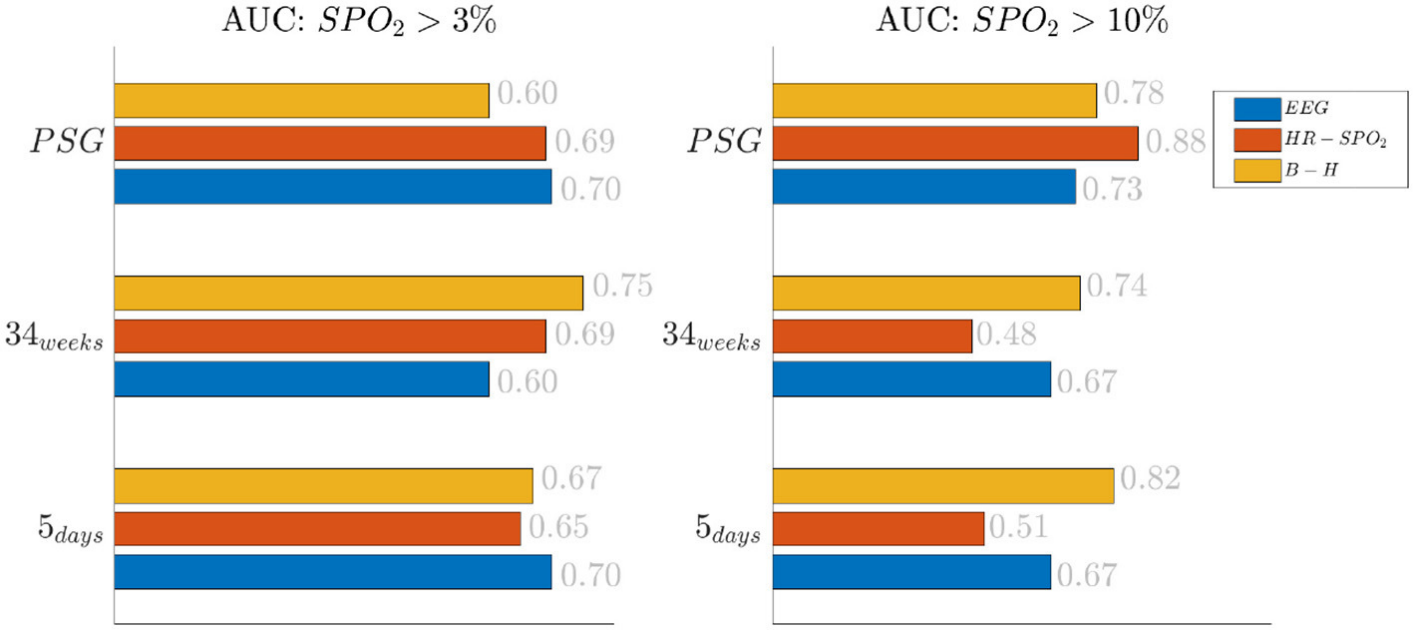
Results of the bradycardia-based classification in three main datasets. The figure here reports the results based on the different feature groups. The left panel reports the area under the curve for desaturations >3%, while the right panel report the results for desaturation >10%. The three colors represent different feature groups: EEG stands for EEG features, HR-*SpO*_2_ represent the cardiovascular features and B-H is related to the brain-heart connectivity.

The effect of the threshold is also reported in Figure 2, where the classification results are shown based on the different feature groups. The left panel shows the AUC for a 3% desaturation threshold, while the right panel shows results for the 10% threshold. The feature group are respectively indicated with the labels EEG, HR *SpO*_2_ and BH for the EEG features, the cardiorespiratory features and the brain-heart features. In the 5 days group and 34 weeks group, either the brain-heart features or the EEG features outperform the HR-*SpO*_2_ group. In addition, the desaturation threshold seems to increase the AUC for the brain-related attributes. On the contrary, the performance seems to be comparable for all different groups at PSG and the effect of the threshold is equally beneficial for the three groups.

**Figure 3:**
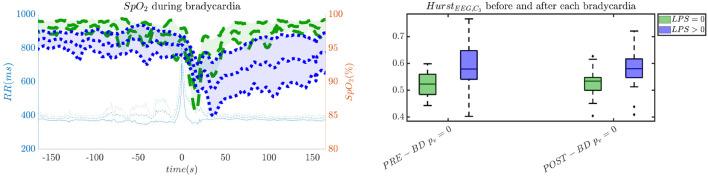
The desaturation levels and the EEG regularity are more pronounced in case of stress. The left panel reports the *SpO*_2_ during the bradycardic spell and the right panel shows the boxplot for the Hurst exponent of channel *C*_3_ for the period before and after each bradycardia. The data are reported for the 5 days group. All the events with a desaturation >10% were included in this figure. The *p*-values in the boxplot are derived via the Kruskal-Wallis test.

**Figure 4:**
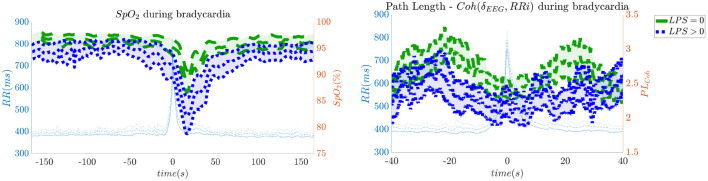
The desaturation levels and the connectivity between delta oscillations and the heart-rate are more pronounced in case of stress. The left panel reports the *SpO*_2_ during the bradycardic spell and the right panel shows the path length derived from the network with EEG channels and the HRV. It is important to remind that the lower the path length, the higher the connectivity. The data are reported for the 34 weeks group. All the events with a desaturation >10% were included in this figure.

**Figure 5:**
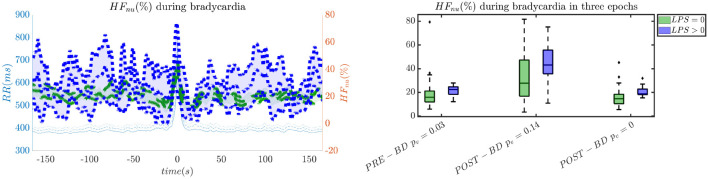
The intensity of bradycardias and the parasympathetic activity are more pronounced in case of stress. The left panel reports the normalized HRV power in the HF band and the right panel shows the normalized power in boxplots before, during and after each bradycardic event. The data are reported for the PSG group. All the events with a desaturation >10% were included in this figure. The *p*-values in the boxplot are derived via the Kruskal-Wallis test.

In order to give an idea of the selected features or the most discriminative information for stress classification, Figures 3–5 reported either the behavior of the selected time-series or the boxplots of the most-discriminative features in epochs before, during and after each bradycardia for the three different monitoring groups. Figure 3 reports the desaturation charts for the 5 days group with *LPS* > 0 (in blue) and *LPS* = 0 (in green) on the left panel, while the Hurst regularity is reported in the period before and after each bradycardia for a 10% threshold on *SpO*_2_. The Hurst exponent shows a higher regularity in case of stress and the *SpO*_2_ charts show higher desaturation in case of stress. Figure 4 reports the desaturation charts and the path length among EEG channels and HRV in the LF band for the 34 weeks group with a 10% threshold on *SpO*_2_. Results reveal a higher desaturation in case of stress as well as a stronger association between the tachogram and the delta-oscillations of the EEG. It is important to remember that the lower the path length, the higher the connectivity. Figure 5 reports the normalized power in the HF band both as time-series and as boxplots for the PSG group with a 10% threshold on *SpO*_2_. The figure does not only suggest a higher and more intense bradycardic spell, but also a more variable bradycardia.

## 4. Discussion

The current study examines the relationship between bradycardic spells and stress burden in premature infants and suggests that stress load can enhance the desaturation and the bradycardic effects. Two novel findings can be reported.

First, this research supports the feasibility of the automatic stress classification based on the physiological reactivity in bradycardias. Levy has shown how routine contact in the NICU could induce respiratory events, such as apneas and hypoapneas, and long oxygen desaturations (Levy et al., 2017). This result has been confirmed by the classification performance reported in Figures 1, 2 and the desaturation charts displayed in Figures 4, 5. The definition of routine handling by Levy et al. follows the notion of stress exposure or procedural pain by Grunau (2013), who defines perinatal stress as accumulation of pain and noxious stimuli. The experienced hands-on-care and pain might trigger a completely different physiological reactivity which could induce a greater desaturation or respiratory burden, as also reported by Levy et al. (2017). Interestingly, the results show a moderate association between the features and the classification outcome (with kappa score between 0.3 and 0.6 for the most of the groups). Although similar studies that perform classification of pain stimuli based on physiological information show strong association between features and the outcome (Brown et al., 2011; Gruss et al., 2015; Misra et al., 2017), it is important to remind that does not elicit any pain in the patient. And yet, it shows that babies experiencing pain the day before the measurement react differently to stress as shown by the stress calculator but also by looking at individual parameters like the desaturation chart, Hurst exponent of the EEG and the HRV in the LF and HF bands.

Second, hypoxic events can impact brain homeostasis. Sleep fragmentation and sleepiness might result from either hands-on-care (especially in infants, Levy et al., 2017) or from desaturation events (especially in apneic patients, Pitson and Stradling, 1998). Sleep fragmentation is able to impact the daily behavior of both adult and NICU patients and is commonly considered a category of pain scoring (Grunau, 2013). Interestingly, Pitson and Stradling (1998) did not only show that the sleepiness and desaturation loads are related in apneic patients, but *SpO*_2_ appears to be related to heart-rate and EEG arousals, intended as increases in frequency. These EEG arousals can be seen in the increase of EEG regularity (Figure 3), while the relationships among *SpO*_2_ dips, heart-rate and EEG arousals might support the higher connectivity between EEG and HRV in the 34 weeks group (Figure 4). In adults, those physiological fingerprints might be the sign of an altered cardiovascular control (Jurysta et al., 2006) or disrupted emotional regulation by the prefrontal cortex (Beebe and Gozal, 2002). Based on these results, one might speculate a possible impact on the brain development and the autonomic regulation development of those infants. However, the exact mechanisms responsible for those events remain still unclear even in adults and further research is still required.

The increase of EEG regularity and desaturation is normally a feature of the first two monitoring groups (Figures 3, 4), while the PSG group is characterized by a greater vagal activity in case of stress exposure (Figure 5). Furthermore, Figures 1, 2 show better classification performance for the PSG data. One might speculate that the effect of stress on the patients' physiology might be easier to discriminate due a lower apnea - bradycardia burden with increasing age and the overall maturation of the ANS (Curzi-Dascalova, 1994). The autonomic development can also explain the increase in performance of cardiovascular features (*HR* − *SPO*_2_) at PSG, while the dominant features are EEG and BH in the first two recording groups (Figure 2, Second Panel). It seems that stress initiates a desaturation effect and regular EEG patterns in the first days of life, while the stress-related HRV patterns only arise at full-term age with the maturation of ANS. It is possible that regular EEG patterns are especially present at younger age because of enhanced hypoxia by hands-on-care (Levy et al., 2017) or a more dysmature EEG. Hypercapnia and reduced cerebral blood flow are common factors to enhance discontinuity of the cerebral activity (West et al., 2006; Weeke et al., 2017). However, the discontinuous EEG might also be triggered by the cumulated pain of the NICU, which increases neonatal burst activity (Slater et al., 2010). In general, dysmature EEG patterns are especially present at younger age and any EEG disruption might be the consequence of subtle effects that can impact the later-life outcome (Watanabe et al., 1999). This relationship between regularity and dysmaturity might further support the hypothesis of an effect on brain development due to enhanced desaturation and exposure to stress.

Similarly to Lin et al. (2016), the interaction between the EEG delta waves showed a strong positive correlation, which increases during the bradycardia spells and under stress exposure (Figure 4). This stronger positive interaction between the slow rhythm of the EEG and the HRV is normally concomitant with a vanishing negative modulation when a sleep state shifts from deep sleep to wake (Bartsch et al., 2015; Lin et al., 2016). This sudden increase in connectivity might indeed be caused due to an underdeveloped parasympathetic control, and the hypoxia event might be considered as a sudden shift toward an awake state. Apneas and other respiratory events are known to lead to sleep fragmentation (Levy et al., 2017) and therefore this increase in connectivity might be a consequence of this sleep disruption. Bartsch et al. (2015) have shown that awake and REM states exhibit stronger physiological connectivity than deep sleep. Especially, the brain-heart interaction increases during REM and awake (Liu et al., 2015). It is possible that the combination of bradycardia and stress exposure might lead the subject to a condition closer to an awake state, with an overall increase of network connectivity.

However, this study has limitations, which have already been considered in the clinical studies by Levy et al. (2017) and Janvier et al. (2004). Bradycardias and apneas are physiological events, whose frequency and severity vary throughout the hospitalization (Janvier et al., 2004). Therefore, there could not be enough events to classify stress levels for the late pre-term, since there are fewer bradycardias and apneas at full-term age. Moreover, the definition of stress or hands-on-care might also influence the design of the classification. Although Levy et al. (2017) pointed out that the clinical handling initiates apneas or hypoapneas, technical contact was also likely to induce desaturations. This study relies on a specific pain scale (LPS), but future research could involve different multidimensional pain scales to have a more in-depth view of the preterm physiology under stress (Jones et al., 2017). The definition of bradycardias or the physiological events of interest might also impact the current analysis. Levy pointed out the different consequences of clinical handling, which does not only include apneas, but also sleep fragmentations, hypoapneas and general desaturation events (Levy et al., 2017). Gee et al. (2017) had a more generic approach, which include all possible bradycardias in his prediction analysis. Specifically, Gee et al. (2017) considered any heart-rate drops for more than 1.2 s as bradycardic event, while Paolillo and Picone (2013) focused only on bradycardias that last for more than 4 s and were concurrent to desaturation events. Based on the fact that the most dangerous de-oxygenation happens with bradycardias (Pichler et al., 2003), the pursued strategy of this investigation focused on events that looked both to desaturations and bradycardias, but it might be possible to reconsider the entire analysis to have only bradycardias. However, the long-term studies on stress aim to quantify the impact on the development of early-life experiences in the NICU and the specific effect of hypoxia was proven detrimental for the development outcome of preterm patients (Janvier et al., 2004). The current study might also be complemented by a longitudinal analysis, using repeated measurement ANOVA or a balanced linear mixed-effect model. However, the current study presents an event-based dataset, where the number of bradycardias vary for each patient and recording time. The number of bradycardias normally reduces with the development of the infant (Curzi-Dascalova, 1994) and the uneven distribution of those events risk to make any within-subject analysis invalid and unrevealing. Therefore, a future study should be designed to monitor bradycardic spell in a longitudinal sense in order to assess whether stress has a persistent effect over the different recordings.

Future steps of this analysis might include a further proof of the development delays in case of apnea load and stress. The multiple attributes derived in this study might be included in a regression model to assess the differences in Bayley scores or other clinical scales (Janvier et al., 2004). Furthermore, the same methodology can be applied to assess the effect of parents-infant interaction with scales, such as the emotional availability scale (Ziv et al., 2000).

In a nutshell, stress seems to induce more intense desaturations, apneic and bradycardic events and cortical activation, which can be the trigger of neurodevelopment impairment. Janvier et al. (2004) have shown how apnea burden can impact the patients' development in terms of cognitive and motor outcome. Pichler et al. (2003) highlighted how long bradycardias can induce severe cerebral deoxygenation in premature infants and Horne et al. (2017) stressed that the cumulated effect of apneas has a long-term negative impact on the cerebral oxygenation of the patients at 5–6 months corrected age. Therefore, an exacerbation of respiratory or hypoxic events due to patient handling or procedural pain can ultimately affect the development of the preterm infants.

## 5. Conclusion

The current study investigated the relationship between stress experience and bradycardias in preterm infants by means of physiological data. Two main findings have been observed. Larger desaturation levels are associated to stress experience. Larger brain-heart synchrony and EEG regularity are observed during hypoxic events linked to procedural pain. The results show that an automatic stress discrimination in premature infants can be implemented assessing the information of the bradycardic spell. In addition, a possible link between stress and neurodevelopment can be envisaged. The enhanced autonomic and hypoxic events we found in stressed infants might impact their frontal cortex activity, which could ultimately affect their developmental outcome. Future research might be required to test this hypothesis.

## Data Availability Statement

The datasets presented in this article are not readily available because the clinical metadata of patient (such as hospital ID, age, recording time-stamps, pain scales) are subject to the European data-privacy policy. Requests to access the datasets should be directed to mlavanga@esat.kuleuven.be. The authors will try to provide an anonymized version of dataset in compliance with the privacy policy of the University Hospitals of Leuven, which is the owner of the data.

## Ethics Statement

The research protocol has been examined and approved by the Ethical Committee of University Hospitals Leuven, Belgium. The study is performed in accordance with the Guidelines for Good Clinical Practice (ICH/GCP) and the latest version of the Declaration of Helsinki. It has been registered at ClinicalTrials.gov (NCT02623400).

## Author Contributions

ML wrote the article and conducted the data analysis. AC and SV supervised the data analysis. BB, KJ, EO, and GN conducted the clinical trial and data collection. BB, KJ, EO, GN, SV, and AC reviewed and corrected the manuscript. All authors contributed to the article and approved the submitted version.

## Conflict of Interest

The authors declare that the research was conducted in the absence of any commercial or financial relationships that could be construed as a potential conflict of interest.
